# Pernicious Pouch of Problems: A Challenging Case of Massive Hemorrhage Secondary to Jejunal Diverticular Bleeding

**DOI:** 10.7759/cureus.74590

**Published:** 2024-11-27

**Authors:** Tyler J Reed, Sarah K Zimmer, Nicholas T Nelson, Allison M Bush

**Affiliations:** 1 Internal Medicine, Naval Medical Center Portsmouth, Portsmouth, USA; 2 Interventional Radiology, Naval Medical Center Portsmouth, Portsmouth, USA; 3 Gastroenterology, Naval Medical Center Portsmouth, Portsmouth, USA

**Keywords:** diverticular bleed, gastrointestinal hemorrhage, gastrointestinal tract, jejunal diverticula, small bowel diverticulosis

## Abstract

Small bowel (SB) diverticulosis is an uncommon diagnosis and a rare cause of gastrointestinal (GI) bleeding. A particularly rare form of SB diverticular disease, jejunal diverticulosis, is usually discovered due to complications, such as hemorrhage, obstruction, or perforation. Owing in part to its rarity, jejunal diverticular bleeding can be difficult to identify and treat, resulting in increased morbidity and mortality. Here, we present the case of a 57-year-old female with recurrent massive GI hemorrhage from a jejunal diverticular vessel that was ultimately diagnosed and successfully managed endoscopically.

## Introduction

Small bowel (SB) diverticulosis is an uncommon condition with an estimated incidence of 0.3-4.5% [1-3]. SB diverticula are typically false diverticula acquired through herniation of the mucosa and submucosa through the muscularis propria. They are classified by anatomical location and denominated as duodenal, jejunal, or ileal [4]. Of these, duodenal diverticulosis is the most common, while jejunal and ileal diverticuloses are particularly rare, together comprising only 18% of cases of SB diverticula [5]. Jejunal diverticulosis (JD) most frequently occurs in males in the sixth and seventh decades of life and is associated with intestinal dysmotility, high intraluminal pressures, and colonic diverticular disease [6]. JD is usually asymptomatic but can present with non-specific symptoms such as abdominal pain, nausea, and malabsorption [6,7]. In 10-30% of cases, JD presents as an acutely complicated disease, such as in cases of perforation, hemorrhage, diverticulitis, or obstruction [4,7]. Due in part to its rarity and location in the small intestine, SB diverticular bleeding can be difficult to identify and treat resulting in prolonged time to diagnosis and increased morbidity and mortality [3]. Here, we present a case of a severe jejunal diverticular hemorrhage with diagnostic and therapeutic challenges.

This article was previously presented as a meeting abstract at the American College of Gastroenterology Annual Scientific Meeting on October 22, 2023.

## Case presentation

**Figure 1:**
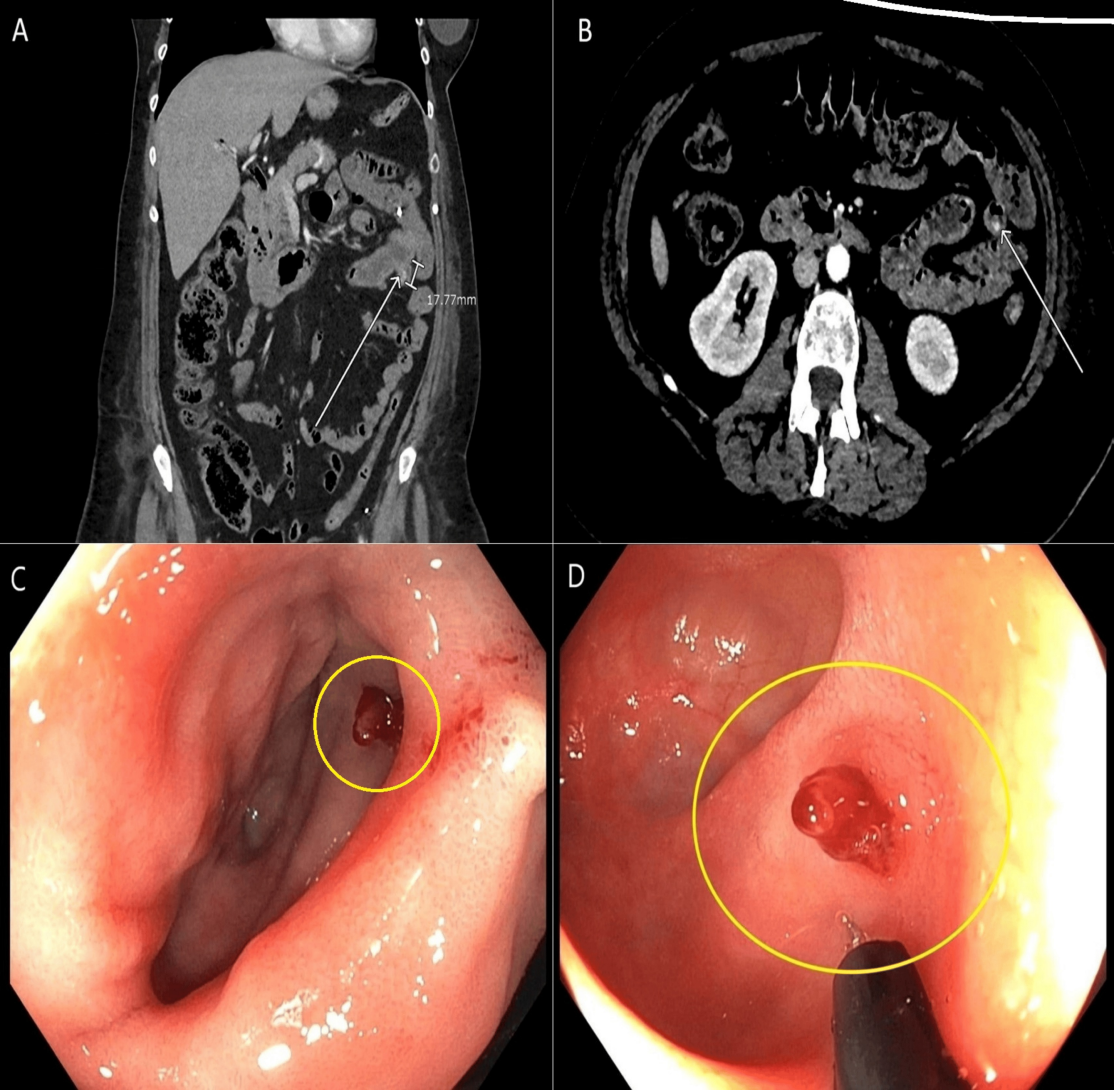
Imaging and endoscopic findings. (A) CTA of the abdomen and pelvis with contrast, coronal view, demonstrating approximately 1.8 cm jejunal diverticulum (white arrow). (B) CTA of the abdomen and pelvis with contrast, axial view, arterial phase, concerning for active extravasation (white arrow). (C, D) EGD and push enteroscopy with a visible vessel inside a large jejunal diverticulum (yellow circles). CTA: computed tomography angiography; EGD: esophagogastroduodenoscopy

A 57-year-old female with a history of splenic artery aneurysm, multiple *Helicobacter pylori* infections, and polymyositis on chronic non-steroidal anti-inflammatory therapy presented to the emergency department after a syncopal episode following two months of progressive dyspnea and exercise intolerance. The syncopal event was preceded by lightheadedness, nausea, and diaphoresis. Initial complete blood cell count (CBC) was notable for a hemoglobin (Hgb) concentration of 10.3 g/dL (reference interval = 11.5-15.5 g/dL), which was lower than the patient’s baseline of 13.2 g/dL in the previous year. Resuscitation with intravenous fluids was initiated, and the patient was admitted for further evaluation. Despite resuscitation with 4 L of crystalloid, the patient continued to demonstrate tachycardia and orthostasis. Approximately 24 hours after admission, the patient experienced large-volume hematemesis and bright red blood per rectum, at which time a repeated CBC revealed a Hgb concentration of 5.5 g/dL, prompting blood product administration, initiation of intravenous pantoprazole, and transfer to the intensive care unit. A computed tomography angiography (CTA) of the abdomen and pelvis was obtained and unrevealing for the bleeding source. Repeated episodes of large-volume hematemesis and hematochezia occurred, and endotracheal intubation was performed for airway protection. Urgent bedside esophagogastroduodenoscopy (EGD) demonstrated SB diverticula but no source of blood loss. After administration of a cumulative eight units of packed red blood cells (pRBCs), two units of fresh frozen plasma (FFP), and one unit of platelets, repeated CBC measurement revealed persistent severe anemia with an Hgb concentration of 4.3 g/dL. Interventional Radiology (IR) performed directed angiography which showed no evidence of arterial extravasation, and a repeated EGD and push enteroscopy again showed multiple SB diverticula without active bleeding, clot, or visible vessels. A colonoscopy was performed and identified no diverticula or active bleeding. Following another episode of large-volume hematochezia, repeated urgent CTA of the abdomen and pelvis demonstrated several diverticula, including a 1.8 cm jejunal diverticula with active arterial bleeding (Figure 1, Panels A, B).

Due to the location of the bleeding vessel and the risk of SB ischemia, IR recommended against repeated directed angiography for embolization. A repeated EGD and push enteroscopy were performed and revealed a large jejunal diverticulum with a non-bleeding visible vessel (Figures 1, Panel C, D), which was treated with epinephrine and placement of three clips. Continuous octreotide infusion was initiated to further control symptoms. Following optimization of medical management and procedural intervention, no additional episodes of hematemesis or hematochezia were observed. The patient was subsequently extubated and continuous infusion therapies were discontinued. Continued clinical stability was noted with a resolution of transfusion requirements. Over the course of this hospitalization, the patient received a total of 19 units of pRBCs, three units of FFP, and one unit of platelets. The patient was subsequently discharged home. At a follow-up appointment one week later, the patient was found to have remained asymptomatic without additional episodes of bleeding and a repeated CBC revealed an Hgb concentration of 11.8 g/dL. Historical Hgb and hematocrit measurements related to this case are displayed in Table 1.

**Table 1:** Hemoglobin and hematocrit trend throughout hospitalization.

	Outpatient baseline	Initial presentation	Status post-hematemesis and bright red blood per rectum	Status post-initial blood product administration	Status post-additional blood product resuscitation and endoscopic management	One week status post-hospitalization	Reference interval
Hemoglobin (g/dL)	13.2	10.3	5.5	4.3	9.0	11.8	11.5–15.5
Hematocrit (%)	39.0	32.1	16.8	13.2	27.6	38.8	34.0–48.0

## Discussion

While diverticulosis is a common etiology of brisk gastrointestinal (GI) bleeding, the vast majority are localized to the colon. Although SB diverticulosis is associated with colonic diverticulosis, SB diverticula are a far less common cause of GI bleeding and impose a much higher risk by comparison due to challenges presented in their diagnosis and treatment. Most often asymptomatic, JD can present as acute complications such as obstruction, diverticulitis, perforation, and bleeding [8]. Severe bleeding from JD, as this case demonstrates, is exceptionally rare with few case reports to its description [8]. One challenge with identifying SB diverticulosis and its complications is in part due to the limited utility of common imaging modalities such as X-ray and abdominal CT [9,10]. Enteroclysis and enterography are available radiographic methods for identifying and attempting localization of jejunal diverticula in clinically stable patients but are not therapeutic. In cases where red cell scintigraphy, barium follow-through studies, and enteroscopy are unable to identify or treat a culprit lesion, diagnostic laparoscopy with partial SB resection may be required [8]. Management of complications from SB diverticula is uniquely challenging due to the anatomy and vasculature. Methods of treating jejunal diverticular bleeding include transcatheter arterial embolization, surgery, and endoscopic therapy. Anatomical location and vascular territory often limit the use of transcatheter arterial embolization due to increased rates of bowel infarction and post-embolization ischemia [11]. Advancements in microcatheter technology with more focused embolization, however, may reduce ischemic bowel injury and other complications [11]. Surgical management with laparotomy and bowel resection remains the dominant strategy for the treatment of jejunal diverticular hemorrhage due to limitations of endoscopic visualization of the small bowel. The use of push enteroscopy as employed in this case, however, has led to an improvement in endoscopic treatment approaches [8,11].

## Conclusions

This case demonstrates a rare, yet potentially life-threatening complication of JD with massive hemorrhage that evaded multiple diagnostic evaluations. Ultimately, a bleeding SB diverticulum was identified radiographically after multiple repeated imaging and endoscopic investigations and was treated endoscopically without recurrence. This case highlights the utility of repeating imaging and endoscopic evaluations in cases of GI bleeding without a clear source on initial evaluations in special consideration of an SB diverticular source. The utilization of an endoscopic management strategy in this case is notable in that similar cases of recurrent SB diverticular bleeding have historically been treated with either surgical resection or angiographic embolization, both of which are associated with an increased risk of SB ischemia in comparison to endoscopic alternatives. This case underscores the necessity of repeated imaging and endoscopic evaluations in identifying SB diverticular bleeding while highlighting the emerging role of endoscopic management as a potentially lower-risk alternative to surgical and angiographic interventions.
